# Contrast-Induced Nephropathy (CIN) After Invasive Treatment of Acute Coronary Syndromes—Predictors, Short and Long-Term Outcome

**DOI:** 10.3390/jcm14113725

**Published:** 2025-05-26

**Authors:** Janusz Sielski, Karol Kaziród-Wolski, Aleksandra M. Piotrowska, Bartłomiej Jurczak, Anna Klasa, Kacper Kozieł, Maciej Ludew, Filip Maj, Lena Merchel, Kamil Pytlak, Michał Zabojszcz, Zbigniew Siudak

**Affiliations:** Institute of Medical Sciences, Collegium Medicum, Jan Kochanowski University, 25-317 Kielce, Poland; jsielski7@interia.pl (J.S.); alemarpiotrowska@gmail.com (A.M.P.); bartekjurczak.68@gmail.com (B.J.); klasa.annamaria@gmail.com (A.K.); s137156@student.ujk.edu.pl (K.K.); maciek.ludew@gmail.com (M.L.); filip.jacek.maj@gmail.com (F.M.); lenamerchel1@gmail.com (L.M.); pytlak.kam@gmail.com (K.P.); michal.zabojszcz@gmail.com (M.Z.); zbigniew.siudak@gmail.com (Z.S.)

**Keywords:** contrast-induced nephropathy, acute coronary syndromes, long-term outcome, kidney disease, vascular access

## Abstract

**Background/Objectives:** Contrast-induced nephropathy (CIN) is a well-documented complication following coronary angiography and percutaneous coronary intervention (PCI). This study aims to evaluate the predictors of CIN and its effect on outcomes in patients with acute coronary syndrome (ACS). **Methods:** A retrospective study included 1579 patients who underwent coronary angiography or PCI. **Results:** The overall incidence of in-hospital CIN was 6.8%, with the highest incidence in the group with eGFR <30 mL/min/1.73 m^2^ at 21.6%. Non-radial vascular access was an independent predictor of CIN occurrence (OR = 2.06 [1.37–3.08]; *p* < 0.001). The risk of death within 30 days was influenced by history of stroke (OR = 4.94 [1.58–15.51]; *p* = 0.006), glucose level on admission (per 10-unit increase) (OR = 1.07 [1.04–1.1]; *p* < 0.001), occurrence of CIN (OR = 5.64 [2.49–12.79]; *p* < 0.001), and hemoglobin level (OR = 0.77 [0.65–0.92]; *p* = 0.003). The risk of death within 365 days was increased by age (OR = 1.05 [1.02–1.07]; *p* < 0.001), history of stroke (OR = 2.45 [1.02–5.89]; *p* = 0.046), glucose levels on admission (per 10-unit increase) (OR = 1.05 [1.03–1.08]; *p* < 0.001), occurrence of CIN (OR = 2.62 [1.42–4.84]; *p* = 0.002), and hemoglobin concentration (OR = 0.78 [0.7–0.88]; *p* = 0.003). An independent predictor of hospitalization for acute or exacerbation of chronic renal failure was baseline creatinine concentration (OR = 3.44 [2.4–4.93]; *p* < 0.001). **Conclusions:** The incidence of CIN is significant, particularly in patients with severe pre-existing renal impairment. Non-radial vascular access is an independent predictor of CIN. The occurrence of CIN is a strong independent predictor of both short-term and long-term mortality.

## 1. Introduction

Cardiovascular disease remains the leading cause of morbidity and mortality worldwide, and coronary artery disease (CHD) represents a significant burden on the global health care system. Among its clinical manifestations, acute coronary syndrome (ACS) is one of the most common causes of emergency hospitalization and invasive treatment. The widespread implementation of coronary angiography and percutaneous coronary interventions (PCI) has significantly improved the prognosis and survival of patients with ACS, allowing rapid revascularization and limiting myocardial damage. However, even these life-saving procedures are not without risk. One well-documented example is in-stent restenosis (ISR), particularly in high-risk subgroups such as patients with diabetes mellitus [1]. Similarly, contrast-induced nephropathy (CIN) remains an important complication, especially in patients undergoing repeated procedures.

CIN is an iatrogenic deterioration of renal function that occurs following the administration of contrast agents used in angiographic procedures [2]. Coronary angiography and PCI are standard procedures in the management of patients with ACS, irrespective of the underlying mechanism of coronary occlusion [3]. Although modern contrast agents have a reduced nephrotoxic effect, the risk of CIN often accumulates with repeated exposures to contrast agents in multiple procedures, alongside various other risk factors, including patient age [4,5]. Several biomarkers and indices are used to predict the risk of CIN, including the renal resistive index and the cardio-ankle vascular index [6,7]. Prevention of CIN, particularly in high-risk patients, remains a critical aspect of management, with adequate hydration before and during the procedure playing a central role [8]. CIN is generally a reversible and transient phenomenon, underscoring the importance of prophylactic measures and appropriate monitoring before discharge [9]. Our study aims to combine an analysis of perioperative factors from the National Registry of Invasive Cardiology Procedures (Ogólnopolski Rejestr Procedur Kardiologii Inwazyjnej, ORPKI) and hospital-specific data from the largest medical center in the Świętokrzyskie province. This study aims to identify contemporary predictors of CIN in patients with ACS treated invasively and to evaluate the impact of CIN on both short-term and long-term prognoses.

## 2. Materials and Methods

### 2.1. Study Population and Data Sources

This single-center retrospective study was conducted using three primary data sources: the ORPKI Registry, hospital records from the Regional Hospital in Kielce, and survival and hospitalization data obtained from the Provincial Branch of the National Health Fund (NFZ). The study protocol was approved by the Bioethics Committee of the Świętokrzyskie Medical Chamber in Kielce (approval number: SIL.BK/1/2023) and was conducted in accordance with the principles of the Declaration of Helsinki. The study population included patients hospitalized for ACS who underwent coronary angiography and/or PCI between 1 January 2015 and 31 December 2018. Clinical data retrieved from hospital records included baseline laboratory parameters such as complete blood counts, serum urea, creatinine, electrolytes, troponin, liver enzymes (alanine aminotransferase and aspartate aminotransferase), glucose, C-reactive protein (CRP), and lipid profile. Additional clinical and angiographic data were obtained from the ORPKI Registry.

### 2.2. Definitions and Procedures

CIN was defined as either a ≥25% relative increase or an absolute increase of ≥0.5 mg/dL (44 μmol/L) in serum creatinine within 48 h after the procedure [10]. Estimated glomerular filtration rate (eGFR) was calculated using the Modification of Diet in Renal Disease (MDRD) equation, which is the standard method used at the study site. Patients were stratified into three groups based on baseline eGFR values: <30 mL/min/1.73 m^2^, 30–60 mL/min/1.73 m^2^, and >60 mL/min/1.73 m^2^. All patients received non-ionic iso-osmolar contrast agents for coronary angiography, including iomeprol (350 mg/dL), iodixanol (270 mg/dL), and iopromide (370 mg/dL). In-hospital management followed the current guidelines of the European Society of Cardiology (ESC) [11,12]. The choice between a radial or non-radial approach depends on the anatomical condition of the arteries in a given case and the current condition of the patient. In some emergency situations (cardiogenic shock), such a choice is originally assumed. The decision on the selection is made by a certified hemodynamic operator. Clinical conditions can affect the difference in the number of types of vascular access presented. These data were not dependent on the authors of the paper. The choice of radial access is associated with lower periprocedural and post-procedural mortality.

### 2.3. Statistical Analysis

Continuous variables were described as mean and standard deviation (SD) or as medians with interquartile ranges, while categorical data were expressed as frequencies and percentages. Comparisons between groups were made using the chi-square test or Fisher’s exact test for categorical variables, and the Mann–Whitney test for continuous, non-normally distributed variables (normality was assessed using the Shapiro–Wilk test). Sample sizes ranging from 500 to 2000 were simulated (1000 iterations per sample size) for varying expected fractions of CIN and levels of estimation precision. Assuming a true CIN incidence between 5% and 15%, a minimum of 1360 subjects is required to estimate the true CIN proportion with a 2% margin of error, 90% statistical power, and 95% confidence. This estimate aligns with recently reported rates of contrast-induced acute kidney injury (CI-AKI), which range from 3.3% to 14.5% [13]. Univariable and multivariable logistic regression models were used to identify factors associated with CIN, 30-day, and 365-day mortality. Odds ratios (OR) with 95% confidence intervals (CI) were calculated. Multivariable analyses employed stepwise backward selection methods. Kaplan–Meier survival curves were constructed, and the log-rank test was used to compare survival across eGFR groups. Statistical significance was set at a two-tailed *p*-value < 0.05. All analyses were performed using R software (version 4.0.3).

## 3. Results

### 3.1. Baseline Characteristics of the Study Population

**Figure 1:**
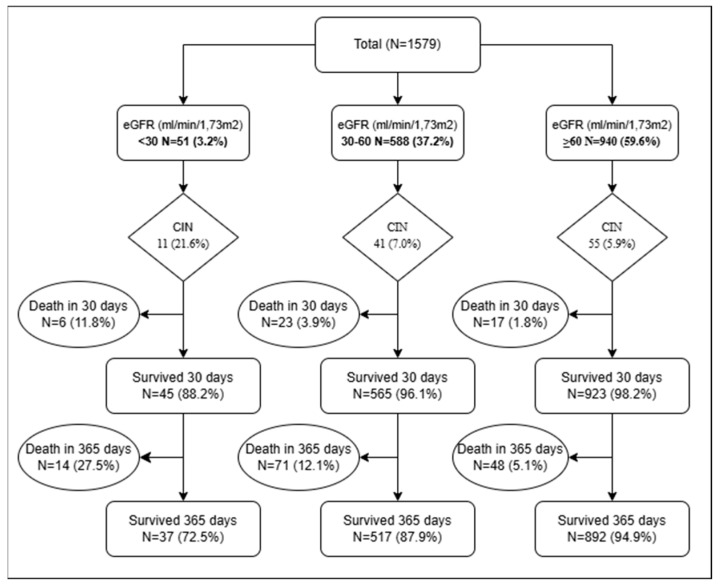
Population of the study. CIN, contrast-induced nephropathy; eGFR, estimated glomerular filtration rate.

**Figure 2:**
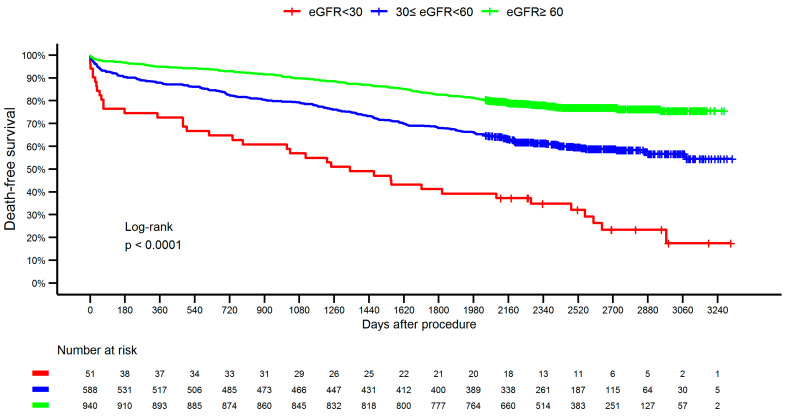
Survival probability according to eGFR group. eGFR, estimated glomerular filtration rate.

A total of 1579 patients with ACS who underwent PCI were included in the analysis. Participants were stratified into three groups based on baseline estimated glomerular filtration rate (eGFR): ≥60, 30–59, and <30 mL/min/1.73 m^2^. Statistically significant differences were observed between the groups with respect to ACS subtype, demographic characteristics (age, sex, weight), comorbidities (diabetes mellitus, hypertension, smoking status), laboratory parameters (urea, glucose, hemoglobin, creatinine), and the incidence of CIN. The subgroup with eGFR <30 mL/min/1.73 m^2^ demonstrated the highest incidence of CIN (21.6%), as well as the highest mortality rates at 30 days (11.8%) and at 365 days (27.5%). This group also had the highest combined rates of death and re-hospitalization at both 30 days (15.7%) and 365 days (47.1%), in addition to the highest rates of hospitalization due to acute renal failure (3.9% at 30 days and 13.7% at 365 days). Descriptive characteristics of the study population are summarized in Table 1. Figure 1 presents the study flowchart, and Figure 2 illustrates the significant differences in long-term survival among the eGFR groups.

### 3.2. Predictors of Contrast-Induced Nephropathy (CIN)

Independent predictor of CIN was only non-radial vascular access, which increased the risk of CIN more than twofold (Table 2).

### 3.3. Predictors of 30-Day Mortality

Factors associated with 30-day mortality included a history of stroke (OR > 4), glucose levels (OR increase of 7% per 10 mg/dL), and the occurrence of CIN (OR > 5). Hemoglobin levels were protective, with each 1 g/dL increase reducing the risk of death by 23% (Table 3).

### 3.4. Predictors of 365-Day Mortality

Long-term mortality (365 days) was associated with age (5% increased risk per year), stroke history (OR > 2), glucose levels (5% increased risk per 10 mg/dL), and CIN (OR > 3). Again, higher hemoglobin levels reduced the risk of death (22% decreased risk per 1 g/dL increase) (Table 4).

### 3.5. Predictors of Rehospitalization Due to Renal Complications

Increased baseline creatinine levels were independently associated with a higher risk of hospitalization for acute or exacerbated chronic renal failure (ICD 10: N18, N19) (OR > 3) (Table 5).

**Figure 3:**
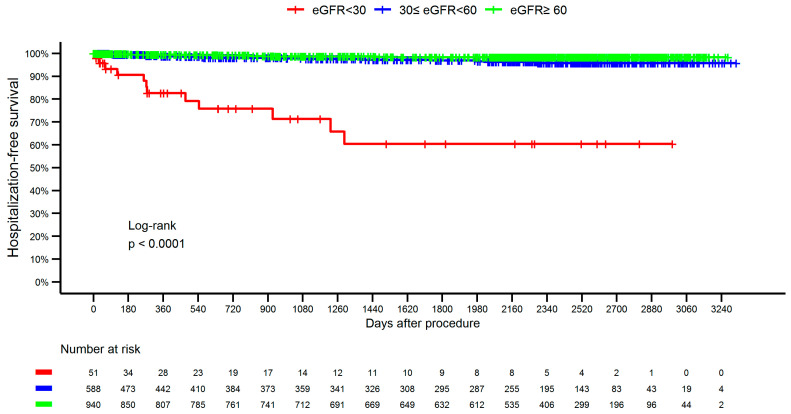
Hospitalization due to acute or chronic renal failure probability according to the eGFR group. eGFR, estimated glomerular filtration rate.

The group with eGFR <30 mL/min/1.73 m^2^ had the highest long-term incidence of hospitalization for acute or chronic renal failure (Figure 3).

**Figure 4:**
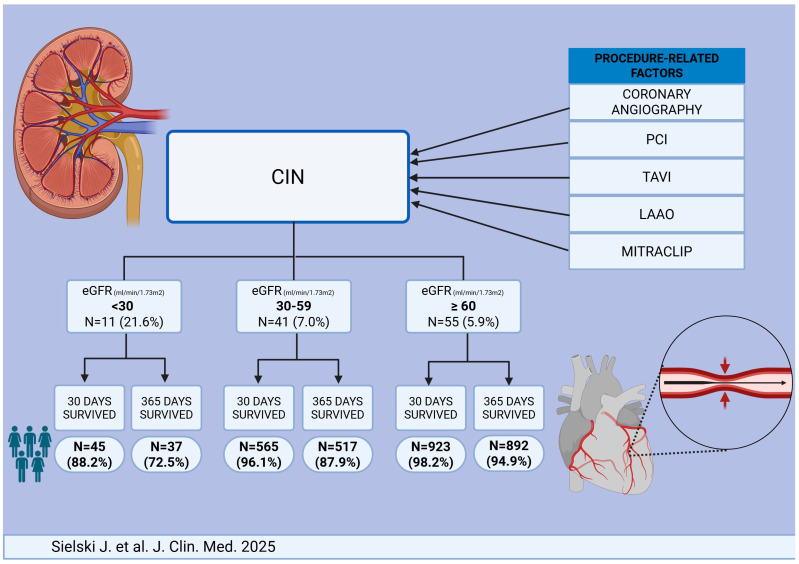
Contrast-Induced Nephropathy (CIN) After Invasive Treatment of Acute Coronary Syndromes—Predictors, Short and Long-Term Outcome.

The summary of findings is shown on the central illustration (Figure 4)

## 4. Discussion

### 4.1. Invasive Cardiology Procedures Requiring the Administration of Contrast Agents

The landscape of invasive cardiology procedures in Poland has been evolving, reflecting both qualitative and quantitative changes over recent years. According to the most recent data from 2022, the number of procedures performed for ST-segment elevation myocardial infarction (STEMI) decreased from 23,684 in 2017 to 17,166 in 2022. Similarly, procedures for non-ST-segment elevation myocardial infarction (NSTEMI) declined from 26,941 to 21,638 over the same period [14]. Despite this decline in the absolute number of ACS interventions, the risk of procedure-related complications, particularly CIN, remains clinically relevant. The observed reduction in ACS interventions may be partially offset by the increasing number of structural heart procedures, such as transcatheter aortic valve implantation (TAVI), MitraClip implantation, and left atrial appendage occlusion (LAAO), which have seen substantial growth in recent years in Poland [14]. Similar to PCI, the periprocedural risk associated with TAVI has been significantly reduced. Nevertheless, CIN remains a considerable concern in these patients and has been linked to increased mortality [15]. A comparable issue has been observed following MitraClip implantation, with postprocedural AKI reported in 29% of patients enrolled in registries [16]. In the context of LAAO procedures, where the baseline risk of renal impairment is elevated due to patient age and the prevalence of multiple risk factors, operators increasingly rely on a combination of echocardiography and contrast fluoroscopy to mitigate renal burden [17].

In our study, the overall incidence of CIN was 6.8%, with the highest rate (21.6%) observed among patients with an eGFR <30 mL/min/1.73 m^2^. The nephrotoxic potential of contrast media has long been a concern for clinicians [18,19,20], and numerous studies have aimed to identify risk factors for CIN.

### 4.2. Risk Factors and Pathophysiology of Contrast-Induced Nephropathy

A review of the literature reveals numerous reports on CIN. Rudnick et al. proposed a CIN risk classification based on baseline eGFR: patients with an eGFR ≥ 45 mL/min/1.73 m^2^ are considered to be at low risk, while those with eGFR < 30 mL/min/1.73 m^2^ are classified as high risk. The intermediate-risk group includes patients with eGFR between 30 and 44 mL/min/1.73 m^2^, particularly individuals with diabetes [21]. These findings support the notion that the risk of CIN is multifactorial and extends beyond renal function alone.

For example, in a prospective single-center study by Sonhaye et al., among 1292 patients undergoing urgent computed tomography (CT) without prior procedural preparation, CIN occurred in 3% of those who received contrast medium [22]. In our cohort of nearly 1600 patients with ACS undergoing PCI, an eGFR < 30 mL/min/1.73 m^2^ was associated with the highest incidence of CIN. Patients who developed CIN also had significantly lower hemoglobin levels (median: 11.4 [10.2–12.7] g/dL) compared to those without CIN (*p* < 0.001), indicating a potential role of anemia as a contributing risk factor. This constellation of factors—lower hemoglobin levels, procedural urgency, and lack of preprocedural optimization—has also been reflected in the risk score proposed by Mehran et al. [23], which includes both anemia (Hgb < 11 g/dL) and clinical factors such as patient condition and emergent presentation as independent predictors of CIN following PCI. It is also worth emphasizing that the risk of CIN is significantly lower in patients undergoing elective procedures who receive appropriate preparation, including assessment of renal function, proper hydration, and optimization of modifiable risk factors. Conversely, in emergency situations where contrast administration is unavoidable and not preceded by prophylactic measures, as was the case in the aforementioned CT study [22], the risk of CIN increases.

In our analysis, the only independent predictor of CIN was non-radial vascular access, which may indirectly reflect the urgent nature of the procedure or the patient’s more severe clinical condition, such as lack of adequate preprocedural preparation, hemodynamic instability, or advanced coronary artery disease. These factors are consistent with the risk model proposed by Mehran et al. [23], in which procedural urgency, hypotension, and the presence of heart failure or other features of advanced disease are identified as key predictors of CIN. Notably, other studies have not confirmed this association [24,25].

### 4.3. Long-Term Outcomes and Prognostic Implications of CIN

Several studies have demonstrated a relationship between ACS and CIN. For instance, Liu et al. reported a CIN incidence of 8.4% in 394 STEMI patients, proposing a CIN risk score incorporating in-hospital mortality and major adverse cardiac events (MACE) [26]. In our analysis, a history of stroke, elevated glucose levels, and the occurrence of CIN were independent risk factors for both 30-day and 365-day mortality, whereas higher hemoglobin levels demonstrated a protective effect across both follow-up periods. The complex and nonlinear relationship between CKD and outcomes after myocardial infarction has been well documented [27,28]. Studies by Liu and Sato have confirmed that CIN is associated not only with short-term complications, but also with long-term renal deterioration and poorer prognosis. Both analyses included a 10-year follow-up of relatively large cohorts: 528 patients with ACS in Liu’s study and 853 patients undergoing contrast-enhanced cardiac investigations in Sato’s study [29,30].

### 4.4. Limitations

Our study has several limitations. It is a retrospective, single-center analysis and lacks data on procedural timing (e.g., symptom onset to balloon), pharmacotherapy, and strategies for CIN management. Notably, the study did not include information on the chronic use of medications such as SGLT-2 inhibitors and statins, which are known to exert nephroprotective effects. SGLT-2 inhibitors administered for at least two weeks prior to coronary intervention have been shown to reduce the risk of CIN in patients with type 2 diabetes mellitus [31]. While all patients at our center received 80 mg of atorvastatin unless contraindicated or already on chronic therapy, the impact of prior statin use was not evaluated in this analysis. Multiple studies have demonstrated the preventive role of statins [32,33], and current guidelines from the ACC/AHA/SCAI and ESC/EACTS recommend high-intensity statin therapy for CIN prevention [34,35].

Our study also does not assess the use of renal replacement therapy (RRT). Although most cases of CIN are transient and resolve within 2–4 weeks, a small proportion of patients may require dialysis. In our center, CIN prophylaxis in NSTEMI and unstable angina (UA) included intravenous hydration with 0.9% NaCl (1.0–1.5 mL/kg/h) administered 3–12 h before and 6–12 h after the procedure, with a target diuresis >150 mL/hour. For STEMI patients, hydration began at the time of qualification for coronary angiography. In patients with reduced LVEF (<40%), fluid administration was conducted with caution. Neither N-acetylcysteine nor sodium bicarbonate was used routinely, consistent with current clinical recommendations [36]. Lastly, the assessment of contrast exposure was based solely on contrast volume, without consideration of hemodynamic parameters or procedure duration, both of which may influence CIN risk. Conditions such as heart failure or hemodynamic instability are known to exacerbate CIN susceptibility [37,38]. While our study includes a relatively large cohort, the findings warrant confirmation in larger, prospective, multi-center trials.

## 5. Conclusions

The incidence of in-hospital CIN in our cohort was higher than previously reported, particularly among patients with eGFR < 30 mL/min/1.73 m^2^. Non-radial vascular access was identified as the only independent predictor of CIN. The occurrence of CIN independently predicted both short-term and long-term mortality. However, in-hospital CIN did not increase the long-term risk of hospitalization for acute or chronic kidney failure.

## Figures and Tables

**Table 1:** Baseline characteristics according to baseline eGFR (based on MDRD, expressed in mL/min/1.73 m^2^).

Variable		<30 (N = 51)	30–59 (N = 588)	≥ 60 (N = 857)	Total (N = 1579)	*p*-Value
Diagnosis n, (%)	NSTEMI	10 (19.6)	87 (14.8)	129 (13.7)	226 (14.3)	0.009
	STEMI	4 (7.8)	58 (9.9)	149 (15.9)	211 (13.4)	
	UA	37 (72.5)	443 (75.3)	662 (70.4)	1142 (72.3)	
Age, year		75 (68–83)	72 (66–79)	63.0 (57–70)	67 (60–75)	<0.001
Male gender n, (%)		19 (37.3)	267 (45.4)	709 (75.4)	995 (63.0)	<0.001
Weight, kg		72 (64.5–80)	76 (67.8–85)	80.0 (70–90)	79 (69–89)	<0.001
Diabetes mellitus n, (%)		17 (33.3)	119 (20.2)	142 (15.1)	278 (17.6)	<0.001
Contrast volume, mL		100 (80–179.5)	110 (80–190)	120 (80–200)	120 (80–200)	0.25
Contrast volume, ml per kg		1.6 (1.1–2.8)	1.6 (1.1–2.4)	1.6 (1.0–2.4)	1.6 (1.1–2.4)	0.78
Previous stroke n, (%)		3 (5.9)	21 (3.6)	27 (2.9)	51 (3.2)	0.31
Previous MI n, (%)		17 (33.3)	147 (25.0)	209 (22.2)	373 (23.6)	0.12
Previous PCI n, (%)		12 (23.5)	135 (23.0)	174 (18.5)	321 (20.3)	0.09
Previous CABG n, (%)		7 (13.7)	39 (6.6)	47 (5)	93 (5.9)	0.03
Smoking status n, (%)		5 (9.8)	72 (12.2)	211 (22.4)	288 (18.2)	<0.001
Arterial hypertension n, (%)		40 (78.4)	433 (73.6)	637 (67.8)	1110 (70.3)	0.03
COPD n, (%)		0 (0.0)	1 (0.2)	0 (0)	1 (0.1)	0.46
Vascular access n, (%)	Radial	21 (43.8)	384 (67.4)	674 (74.2%)	1079 (70.7)	<0.001
	other	27 (56.2%)	186 (32.6%)	234 (25.8%)	447 (29.3%)	
Urea, mg/dl		81 (64–108.5)	46 (37–57)	35 (29–41)	38 (31–49)	<0.001
Glucose level on admission, md/dL		139.0 (96.5–211.5)	116.0 (94–158.8)	110.0 (95–142)	112.0 (95–150)	0.007
Hemoglobin, G/dL		11.4 (10.2–12.7)	13.3 (12.2–14.4)	14.2 (13.1–15.1)	13.9 (12.6–14.8)	<0.001
Hematocrit (%)		33.7 (31.5–37.8)	39.4 (36.3–42.9)	41.8 (38.8–44)	40.8 (37.4–43.7)	<0.001
Red blood count, T/L		3.8 (3.4–4.2)	4.4 (4.1–4.8)	4.7 (4.3–5)	4.6 (4.2–4.9)	<0.001
White blood cells, G/L		7.8 (6.2–10.7)	8.3 (6.8–10.4)	8.2 (6.8–10.1)	8.2 (6.8–10.3)	0.59
Platelets, G/L		222 (183.5–258)	215 (179–257)	225.0 (188–268.5)	222 (185–264)	0.009
Troponin T hs, ng/L		111.7 (52.7–469.3)	31.1 (11.7–172.7)	31.6 (9.5–178.3)	34 (10.6–178.3)	<0.001
Total cholesterol, md/dL		165.5 (127.5–196)	171.0 (139–207)	178.0 (149–215)	175 (145–211)	0.003
HDL cholesterol, md/dL		40.0 (31.8–46)	43 (36–52)	43 (36–51)	43 (36–51)	0.05
Non-HDL cholesterol, md/dL		130.5 (94.5–159.2)	126 (96–162)	135.0 (107.5–168)	130 (102–165)	0.003
LDL cholesterol, md/dL		94 (71–118.5)	101.5 (73–135)	109.0 (83–141)	106 (77–138)	0.001
Triglicerydes, md/dL		136 (101–185)	120 (91–162)	118.0 (89–159)	120 (90–161)	0.31
ALT, U/L		21 (15–35.5)	24.0 (17–34)	26.0 (18–37)	25 (18–36)	0.02
AST, U/L		28.5 (21.5–51.8)	28.0 (22–41)	29.0 (22–42)	28 (22–42)	0.95
Sodium, mmol/L		139 (136.5–142)	139 (138, 141)	140.0 (138–141)	140 (138–141)	0.43
Potassium, mmol/L		4.7 (4.3–5.4)	4.3 (4.0–4.6)	4.3 (4–4.6)	4.3 (4.0–4.6)	<0.001
C-reactive protein, mg/L		7.7 (2.4–22.4)	5.1 (1.9–14.1)	4.8 (1.7–19.4)	5.0 (1.8–18.3)	0.62
Baseline eGFR, ml/min/1.73 m^2^		24.1 (15.6–28.2)	51.1 (44.8–56)	71.9 (66–79.8)	63.9 (52.7–73.8)	<0.001
Baseline creatinine [mg/dL]		2.2 (1.8–3.6)	1.2 (1.1–1.4)	1.0 (0.9–1.1)	1.0 (0.9–1.2)	<0.001
CIN n, (%)		11 (21.6%)	41 (7.0%)	55 (5.9%)	107 (6.8%)	<0.001
Death or hospitalization in 30 days n, (%)		8 (15.7%)	33 (5.6%)	32 (3.4%)	73 (4.6%)	0.001
Death in 30 days n, (%)		6 (11.8%)	23 (3.9%)	17 (1.8%)	46 (2.9%)	<0.001
Death or hospitalization in 365 days n, (%)		24 (47.1%)	151 (25.7%)	135 (14.4%)	310 (19.6%)	<0.001
Death in 365 days n, (%)		14 (27.5%)	71 (12.1%)	48 (5.1%)	133 (8.4%)	<0.001
Hospitalization due to acute or chronic kidney disease in 30 days, n, (%)		2 (3.9%)	0 (0.0%)	1 (0.1%)	3 (0.2%)	0.003
Hospitalization due to acute or chronic kidney disease in 365 days, n, (%)		7 (13.7%)	6 (1.0%)	5 (0.5%)	18 (1.1%)	<0.001

ALT, alanine aminotransferase; AST, aspartate aminotransferase; CABG, coronary artery bypass grafting; CIN, contrast-induced nephropathy; COPD, chronic obstructive pulmonary disease; eGFR, estimated glomerular filtration rate; HDL, high-density lipoprotein; LDL, low-density lipoprotein; MI, myocardial infarction; NSTEMI, non-ST-elevation myocardial infarction; PCI, percutaneous coronary intervention; STEMI, ST-elevation myocardial infarction; UA, unstable angina.

**Table 2:** Factors affecting the occurrence of contrast-induced nephropathy.

Variable		Univariable		Multivariable (FORWARD)	
		OR (95% CI)	*p*-Value	OR (95% CI)	*p*-Value
Diagnosis	NSTEMI	Ref. level		NA	
	STEMI	1.36 (0.62–2.99)	0.44	NA	
	UA	1.34 (0.72–2.51)	0.35	NA	
Age, per year		1.03 (1.01–1.05)	0.007	NA	
Gender [male]		0.86 (0.58–1.29)	0.48	NA	
Weight [kg]		0.99 (0.98–1)	0.18	NA	
Diabetes mellitus [yes]		1.47 (0.92–2.35)	0.11	NA	
Contrast volume per 10 mL increase		1.75 (0.34–8.98)	0.50	NA	
Previous stroke [yes]		1.88 (0.78–4.52)	0.16	NA	
Previous MI [yes]		1.1 (0.7–1.73)	0.69	NA	
Previous PCI [yes]		0.89 (0.54–1.48)	0.66	NA	
Previous CABG [yes]		0.95 (0.4–2.22)	0.90	NA	
Smoking status [yes]		0.9 (0.53–1.52)	0.69	NA	
Arterial hypertension [yes]		0.9 (0.59–1.37)	0.63	NA	
COPD [yes]		NA		NA	
Vascular access	Radial	Ref. level		Ref. level	
	other	2.06 (1.37–3.08)	<0.001	2.06 (1.37–3.08)	<0.001
Urea [mg/dl]		1.02 (1.01–1.02)	<0.001	NA	
Glucose level on admission, per 10 units increase [md/dL]		1.03 (1–1.05)	0.02	NA	
Hemoglobin (g/dL)		0.78 (0.7–0.87)	<0.001	NA	
Hematocrit (%)		0.92 (0.88–0.95)	<0.001	NA	
Red blood count (T/L)		0.47 (0.35–0.64)	<0.001	NA	
White blood cells (G/L)		1.01 (0.99–1.03)	0.3	NA	
Platelets, per 25 units increase (G/L)		0.98 (0.91–1.05)	0.58	NA	
Troponin T hs per 100 units increase (ng/L)		1.02 (1–1.04)	0.02	NA	
Total cholesterol per 10 units increase [md/dL]		1 (0.96–1.04)	0.87	NA	
HDL cholesterol [md/dL]		0.99 (0.97–1.01)	0.27	NA	
Non-HDL cholesterol per 10 units increase [md/dL]		1 (0.96–1.04)	0.96	NA	
LDL cholesterol per 10 units increase [md/dL]		1 (0.95–1.05)	0.96	NA	
Triglicerydes per 10 units increase [md/dL]		1.01 (0.99–1.03)	0.25	NA	
ALT per 5 units increase [U/L]		1.01 (1–1.02)	0.03	NA	
AST per 5 units increase [U/L]		1.01 (1–1.02)	0.009	NA	
Sodium [mmol/L]		0.94 (0.89–1)	0.06	NA	
Potassium [mmol/L]		1.5 (0.99–2.25)	0.05	NA	
C-reactive protein [mg/L]		1.01 (1–1.01)	<0.001	NA	
Baseline eGFR [mL/min/1.73 m^2^]		1 (0.99–1.01)	0.95	NA	
Baseline eGFR per 5 units [mL/min/1.73 m^2^]		1 (0.94–1.06)	0.95	NA	
Baseline eGFR [mL/min/1.73 m^2^]	≥60	Ref. level		NA	
	30–60	1.21 (0.79–1.83)	0.38	NA	
	<30	4.42 (2.15–9.1)	<0.001	NA	
Baseline creatinine [mg/dL]		1.64 (1.29–2.1)	<0.001	NA	

ALT, alanine aminotransferase; AST, aspartate aminotransferase; CABG, coronary artery bypass grafting; COPD, chronic obstructive pulmonary disease; eGFR, estimated glomerular filtration rate; HDL, high-density lipoprotein; LDL, low-density lipoprotein; MI, myocardial infarction; NSTEMI, non-ST-elevation myocardial infarction; PCI, percutaneous coronary intervention; STEMI, ST-elevation myocardial infarction; UA, unstable angina.

**Table 3:** Factors affecting death in 30 days.

Variables		Univariable		Multivariable (FORWARD)	
		OR (95% CI)	*p*-Value	OR (95% CI)	*p*-Value
Diagnosis	NSTEMI	Ref. level		NA	
	STEMI	0.61 (0.24–1.58)	0.31	NA	
	UA	0.43 (0.22–0.87)	0.02	NA	
Age, per year		1.03 (1.01–1.05	<0.001	NA	
Gender [male]		0.83 (0.46–1.51)	0.54	NA	
Weight [kg]		0.98 (0.96–1)	0.04	NA	
Diabetes mellitus [yes]		0.7 (0.29–1.66)	0.41	NA	
Contrast volume per 10 mL increase		1.07 (0.08–14.06)	0.96	NA	
Previous stroke [yes]		4.96 (2–12.3)	<0.001	4.94 (1.58–15.51)	0.006
Previous MI [yes]		1.28 (0.67–2.47)	0.45	NA	
Previous PCI [yes]		0.58 (0.24–1.38)	0.22	NA	
Previous CABG [yes]		0.72 (0.17–3.02)	0.65	NA	
Smoking status [yes]		0.94 (0.43–2.04)	0.88	NA	
Arterial hypertension [yes]		1.2 (0.62–2.34)	0.59	NA	
COPD [yes]		NA		NA	
Vascular access	Radial	Ref. level		NA	
	other	2.36 (1.27–4.4)	0.007	NA	
Urea [mg/dL]		1.03 (1.01–1.04)	<0.001	NA	
Glucose level on admission, per 10 units increase [md/dL]		1.06 (1.04–1.09)	<0.001	1.07 (1.04–1.1)	<0.001
Hemoglobin [g/dl]		0.76 (0.65–0.88)	<0.001	0.77 (0.65–0.92)	0.003
Hematocrit (%)		0.91 (0.86–0.96)	<0.001	NA	
Red blood count (T/L)		0.5 (0.32–0.78)	0.003	NA	
White blood cells (G/L)		1.02 (1–1.03)	0.06	NA	
Platelets, per 25 units increase (G/L)		0.91 (0.81–1.02)	0.12	NA	
Troponin T hs per 100 units increase (ng/L)		1.04 (1.02–1.05)	<0.001	NA	
Total cholesterol per 10 units increase [md/dL]		0.9 (0.83–0.96)	0.002	NA	
HDL cholesterol [md/dL]		0.97 (0.94–1)	0.07	NA	
Non-HDL cholesterol per 10 units increase [md/dL]		0.91 (0.85–0.98)	0.02	NA	
LDL cholesterol per 10 units increase [md/dL]		0.91 (0.84–0.99)	0.02	NA	
Triglicerydes per 10 units increase [md/dL]		0.99 (0.96–1.04)	0.80	NA	
ALT per 5 units increase [U/L]		1.02 (1.01–1.04)	0.004	NA	
AST per 5 units increase [U/L]		1.03 (1.01–1.04)	<0.001	NA	
Sodium [mmol/L]		0.84 (0.78–0.9)	<0.001	NA	
Potassium [mmol/L]		1.53 (0.84–2.8)	0.17	NA	
C-reactive protein [mg/L]		1.01 (1–1.02)	<0.001	NA	
Baseline eGFR [mL/min/1.73 m^2^]		0.97 (0.95–0.98)	<0.001	NA	
Baseline eGFR per 5 units [mL/min/1.73 m^2^]		0.85 (0.78–0.92)	<0.001	NA	
Baseline eGFR [mL/min/1.73 m^2^]	≥60	Ref. level		NA	
	30–60	2.21 (1.17–4.17)	0.01	NA	
	<30	7.24 (2.72–19.24)	<0.001	NA	
Baseline creatinine [mg/dL]		1.64 (1.29–2.1)	<0.001	NA	
CIN [yes]		6.77 (3.49–13.13)	<0.001	5.64 (2.49–12.79)	<0.001

ALT, alanine aminotransferase; AST, aspartate aminotransferase; CABG, coronary artery bypass grafting; CIN, contrast-induced nephropathy; COPD, chronic obstructive pulmonary disease; eGFR, estimated glomerular filtration rate; HDL, high-density lipoprotein; LDL, low-density lipoprotein; MI, myocardial infarction; NSTEMI, non-ST-elevation myocardial infarction; PCI, percutaneous coronary intervention; STEMI, ST-elevation myocardial infarction; UA, unstable angina.

**Table 4:** Factors affecting death in 365 days.

Variables		Univariable		Multivariable (FORWARD)	
		OR (95% CI)	*p*-Value	OR (95% CI)	*p*-Value
Diagnosis	NSTEMI	Ref. level		Ref. level	
	STEMI	0.56 (0.29–1.09)	0.09	NA	
	UA	0.75 (0.4–1.01)	0.05	NA	
Age [years]		1.07 (1.05–1.09)	<0.001	1.05 (1.02–1.07)	<0.001
Gender [male]		1.01 (0.7–1.45)	0.97	NA	
Weight [kg]		0.97 (0.96–0.99)	<0.001	NA	
Diabetes mellitus [yes]		0.98 (0.61–1.56)	0.92	NA	
Contrast volume per 10 mL increase		1.88 (0.43–8.26)	0.40		
Previous stroke [yes]		2.43 (1.15–5.1)	0.02	2.45 (1.02–5.89)	0.046
Previous MI [yes]		1.03 (0.68–1.56)	0.90	NA	
Previous PCI [yes]		0.59 (0.35–0.99)	0.04	NA	
Previous CABG [yes]		0.47 (0.17–1.31)	0.15	NA	
Smoking status [yes]		0.79 (0.56–1.1)	0.45	NA	
Hypertension [yes]		1.06 (0.81–1.4)	0.62	NA	
COPD [yes]		NA		NA	
Vascular access	Radial	Ref. level		NA	
	other	1.51(1.03–2.21)	0.03	NA	
Urea [mg/dL]		1.03 (1.02–1.04)	<0.001	NA	
Glucose level on admission, per 10 units increase [md/dL]		1.05 (1.03–1.07)	<0.001	1.05 (1.03–1.08)	<0.001
Hemoglobin [g/dL]		0.72 (0.65–0.79)	<0.001	0.78 (0.7–0.88)	<0.001
Hematocrit (%)		0.9 (0.87–0.93)	<0.001	NA	
Red blood count (T/L)		0.41 (0.31–0.55)	<0.001	NA	
White blood cells (G/L)		1.02 (1–1.04)	0.09	NA	
Platelets, per 25 units increase (G/L)		0.96 (0.9–1.03)	0.28	NA	
Troponin T hs per 100 units increase (ng/L)		1.03 (1.01–1.04)	<0.001	NA	
Total cholesterol per 10 units increase [md/dL]		0.93 (0.89–0.96)	<0.001	NA	
HDL cholesterol [md/dL]		0.98 (0.97–1.0)	0.03	NA	
Non-HDL cholesterol per 10 units increase [md/dL]		0.93 (0.9–0.98)	0.002	NA	
LDL cholesterol per 10 units increase [md/dL]		0.94 (0.9–0.98)	0.006	NA	
Triglicerydes per 10 units increase [md/dL]		0.96 (0.93–0.99)	0.02	NA	
ALT per 5 units increase [U/L]		1.01 (1–1.02)	0.008	NA	
AST per 5 units increase [U/L]		1.01 (1–1.02)	0.001	NA	
Sodium [mmol/L]		0.89 (0.85–0.94)	<0.001	NA	
Potassium [mmol/L]		1.46 (1–2.12)	0.046	NA	
C-reactive protein [mg/L]		1.01 (1.01–1.01)	<0.001	NA	
Baseline eGFR [mL/min/1.73 m^2^]		0.97 (0.96–0.98)	<0.001	NA	
Baseline eGFR [mL/min/1.73 m^2^]	≥60	Ref. level		NA	
	30–59	2.55 (1.74–3.74)	<0.001	NA	
	<30	7.03 (3.56–13.88)	<0.001	NA	
Baseline creatinine [mg/dL]		1.77 (1.38–2.27)	<0.001	NA	
CIN [yes]		3.85 (2.36–6.28)	<0.001	2.62 (1.42–4.84)	0.002

ALT, alanine aminotransferase; AST, aspartate aminotransferase; CABG, coronary artery bypass grafting; CIN, contrast-induced nephropathy; COPD, chronic obstructive pulmonary disease; eGFR, estimated glomerular filtration rate; HDL, high-density lipoprotein; LDL, low-density lipoprotein; MI, myocardial infarction; NSTEMI, non-ST-elevation myocardial infarction; PCI, percutaneous coronary intervention; STEMI, ST-elevation myocardial infarction; UA, unstable angina.

**Table 5:** Factors affecting hospitalization due to acute kidney injury or chronic kidney disease in 365 days.

		Univariable		Multivariable	
Variables		OR (95% CI)	*p*-Value	OR (95% CI)	*p*-Value
Diagnosis	NSTEMI	Ref. level		NA	
	STEMI	0.53 (0.1–2.93)	0.47	NA	
	UA	0.59 (0.19–1.84)	0.36	NA	
Age, per year		1 (0.96–1.04)	0.90	NA	
Gender [male]		2.07 (0.68–6.32)	0.20	NA	
Weight [kg]		0.97 (0.94–1)	0.06	NA	
Diabetes mellitus [yes]		1.34 (0.44–4.11)	0.61	NA	
Contrast volume per 10 mL/kg increase		7.18 (0.27–191.82)	0.24	NA	
Previous stroke [yes]		NA		NA	
Previous MI [yes]		1.25 (0.44–3.52)	0.68	NA	
Previous PCI [yes]		0.78 (0.22–2.72)	0.70	NA	
Previous CABG [yes]		NA		NA	
Smoking status [yes]		1.28 (0.42–3.93)	0.66	NA	
Arterial hypertension [yes]		0.66 (0.25–1.71)	0.39	NA	
COPD [yes]		NA		NA	
Vascular access	Radial	Ref. level		NA	
	other	1.7 (0.64–4.5)	0.28	NA	
Urea [mg/dL]		1.04 (1.02–1.05)	<0.001	NA	
Glucose, per 10 units increase [md/dL]		1 (0.93–1.07)	0.97	NA	
Hemoglobin [g/dL] (G/dL)		0.64 (0.52–0.79)	<0.001	NA	
Hematocrit (%)		0.86 (0.8–0.93)	<0.001	NA	
Red blood count (T/L)		0.26 (0.14–0.49)	<0.001	NA	
White blood cells (G/L)		0.83 (0.67–1.02)	0.08	NA	
Platelets, per 25 units increase (G/L)		1.03 (0.89–1.2)	0.68	NA	
Troponin T hs per 100 units increase (ng/L)		1 (0.94–1.06)	0.90	NA	
Total cholesterol per 10 units increase [md/dL]		0.94 (0.84–1.04)	0.23	NA	
HDL cholesterol [md/dL]		0.95 (0.91–1)	0.06	NA	
Non-HDL cholesterol per 10 units increase [md/dL]		0.95 (0.85–1.06)	0.40	NA	
LDL cholesterol per 10 units increase [md/dL]		0.93 (0.82–1.05)	0.24	NA	
Triglicerydes per 10 units increase [md/dL]		1.02 (0.97–1.06)	0.46	NA	
ALT per 5 units increase [U/L]		0.82 (0.65–1.02)	0.07	NA	
AST per 5 units increase [U/L]		0.95 (0.85–1.06)	0.39	NA	
Sodium [mmol/L]		0.95 (0.82–1.09)	0.46	NA	
Potassium [mmol/L]		4.63 (2.12–10.13)	<0.001	NA	
C-reactive protein [mg/L]		0.99 (0.97–1.02)	0.58	NA	
Baseline eGFR [mL/min/1.73 m^2^]		0.92 (0.9–0.95)	<0.001	NA	
Baseline eGFR per 5 units [mL/min/1.73 m^2^]		0.67 (0.59–0.77)	<0.001	NA	
Baseline eGFR [mL/min/1.73 m^2^]	≥60	Ref. level		NA	
	30–60	1.93 (0.59–6.35)	0.28	NA	
	<30	29.75 (9.08–97.48)	<0.001	NA	
Baseline creatinine [mg/dL]		3.44 (2.4–4.93)	<0.001	3.44 (2.4–4.93)	<0.001
CIN [yes]		4.04 (1.31–12.51)	0.02	NA	

ALT, alanine aminotransferase; AST, aspartate aminotransferase; CABG, coronary artery bypass grafting; CIN, contrast-induced nephropathy; COPD, chronic obstructive pulmonary disease; eGFR, estimated glomerular filtration rate; HDL, high-density lipoprotein; LDL, low-density lipoprotein; MI, myocardial infarction; NSTEMI, non-ST-elevation myocardial infarction; PCI, percutaneous coronary intervention; STEMI, ST-elevation myocardial infarction; UA, unstable angina.

## Data Availability

The datasets generated during and/or analyzed during the current study are available from the corresponding author on reasonable request.
